# The Effect on Quality of Life of Therapeutic Plasmapheresis and Intravenous Immunoglobulins on a Population of Myalgic Encephalomyelitis/Chronic Fatigue Syndrome Patients with Elevated β-Adrenergic and M3-Muscarinic Receptor Antibodies—A Pilot Study

**DOI:** 10.3390/jcm14113802

**Published:** 2025-05-29

**Authors:** Boglárka Oesch-Régeni, Nicolas Germann, Georg Hafer, Dagmar Schmid, Norbert Arn

**Affiliations:** 1 Division of Nephrology and Transplant Medicine, HOCH Health Eastern Switzerland, Cantonal Hospital St. Gallen, 9000 St. Gallen, Switzerland; 2 Department of Internal Medicine, Cantonal Hospital Münsterlingen, 8596 Münsterlingen, Switzerland; 3 Department of Internal Medicine, HOCH Health Eastern Switzerland, Hospital Linth, 8730 Uznach, Switzerland; nicolas.germann@h-och.ch; 4 Department of Internal Medicine, HOCH Health Eastern Switzerland, Cantonal Hospital St. Gallen, 9000 St. Gallen, Switzerland; georg.hafer@h-och.ch; 5 Division of Psychosomatic Medicine and Consultation-Liaison Psychiatry, HOCH Health Eastern Switzerland, Cantonal Hospital St. Gallen, 9000 St. Gallen, Switzerland; dagmar.schmid@h-och.ch

**Keywords:** ME/CFS, long COVID, post-COVID-19 condition (PCC), postacute sequelae of SARS-CoV-2 infection (PASC), plasmapheresis, IVIG, psychometry, EQ-5D-5L, G protein-coupled receptors, quality of life, microclots, adrenergic β1-receptor antibody, adrenergic β2-receptor antibody, M3-muscarinic acetylcholine receptor antibody, M4-muscarinic acetylcholine receptor antibody

## Abstract

**Background/Objectives:** Myalgic encephalomyelitis/chronic fatigue syndrome (ME/CFS) is a debilitating condition with not fully understood causes, though evidence points to immune system involvement and possible autoimmunity. ME/CFS could be triggered by various infectious pathogens, like SARS-CoV-2; furthermore, a subset of the post-COVID-19 condition (PCC) patients fulfill the diagnostic criteria of ME/CFS. According to the Canadian Consensus Criteria (CCC), the presence of specific symptoms such as fatigue, post-exertional malaise, sleep dysfunction, pain, neurological/cognitive manifestations, and symptoms from at least two of the following categories lead to the diagnosis of ME/CFS: autonomic, neuroendocrine, and immune manifestation. In this study, the patient selection was based on the identification of ME/CFS patients with elevated autoantibodies, regardless of the triggering factor of their condition. **Methods:** The aim of this study was to identify ME/CFS patients among long COVID patients with elevated autoantibodies. In seven cases, plasmapheresis (PE) and intravenous immunoglobulins (IVIGs) with repetitive autoantibody measurements were applied: four PE sessions on days 1, 5, 30, and 60, and a low-dose IVIG therapy after each treatment. Antibodies were measured before the first PE and two weeks after the last PE session. To monitor clinical outcomes, the following somatic and psychometric follow-up assessments were conducted before the first PE, 2 weeks after the second, and 2 weeks after the last PE: the Schellong test, ISI (insomnia), FSS (fatigue), HADS (depression and anxiety), and EQ-5D-5L (quality of life) questionnaires. **Results:** There was a negative association between both the β2-adrenergic and M3-muscarinic receptor autoantibody concentration and the quality of life measurements assessed with the EQ-5D-5L questionnaire. Per 1 U/mL increase in the concentration levels of β2-adrenergic receptor antibodies or M3-muscarinic acetylcholine receptor antibodies, the EQ-5D-5L index score [−0.59 to 1] decreased by 0.01 (0.63%) or 0.02 (1.26%), respectively. There were no significant associations between the ISI, HADS, and FSS questionnaires and the β1-adrenergic and M4-muscarinic receptor antibodies titers. **Conclusions:** After a thorough selection of patients with present autoantibodies, this pilot study found negative associations concerning autoantibody concentration and somatic, as well as psychological wellbeing. To validate these promising feasibility study results—indicating the potential therapeutic potential of antibody-lowering methods—further investigation with larger sample sizes is needed.

## 1. Introduction

The COVID-19 pandemic, caused by the severe acute respiratory syndrome coronavirus 2 (SARS-CoV-2), has led to a global health crisis with profound immediate and long-term consequences. While the acute phase of COVID-19 has been well-documented, increasing attention is being directed toward the persistent, debilitating symptoms experienced by a subset of patients well beyond the initial infection period. These prolonged symptoms fall under the umbrella term post-COVID-19 condition (PCC) or postacute sequelae of SARS-CoV-2 infection (PASC), also referred to as long COVID [1]. Its consensus definition for adults is the following: post-COVID-19 condition occurs in individuals with a history of probable or confirmed SARS-CoV-2 infection, usually three months from the onset, with symptoms that last for at least two months and cannot be explained by an alternative diagnosis. PCC encompasses more than 200 symptoms, the leading and most common ones being fatigue, exertion intolerance, cognitive impairment, orthostatic intolerance (postural orthostatic tachycardia syndrome—POTS), autonomic dysfunction, and muscle and joint pain which can persist for months or even years after the acute infection has been resolved [1].

Epidemiological studies suggest that approximately 5–10% of individuals who recover from the acute phase of COVID-19 develop PCC [2]. The clinical presentation of PCC is highly variable, and a subset of the affected individuals fulfills diagnostic criteria of myalgic encephalomyelitis/chronic fatigue syndrome (ME/CFS) [1,3,4]. The pathophysiological mechanisms underlying PCC are still not completely understood and are complex and multifactorial, involving possible immune dysregulation, autoimmunity, endothelial dysfunction, viral persistence, and the reactivation of latent viruses such as Epstein–Barr virus (EBV) [3]. Additionally, endothelial dysfunction, characterized by inflammation, microclots, and hypoperfusion, has been identified as a significant contributor to the persistent symptoms observed in PCC [5].

Recent research has highlighted the role of immune activation and dysregulation in PCC, with several studies reporting the presence of autoantibodies, including those targeting G-protein-coupled receptors (GPCRs) like the β2-adrenergic receptor [1]. These autoantibodies have been associated with symptom severity and functional impairment in some studies regarding both PCC and ME/CFS [4,6]. It must be mentioned that several studies have stated that elevated concentrations of AABs are not pathognomonic. In a study of 80 subjects, healthy individuals had higher AAB levels compared to patients with ME/CFS and/or PCC, despite being asymptomatic [4]. Although, among the PCC/ME/CFS patients, the concentrations of AABs correlated with symptom severity [4]. Therefore, GPCR antibodies do not seem to be an optimal marker of ME/CFS or PCC, but in affected individuals they could serve as a therapeutic target. The other clinical condition, ME/CFS is—according to the revised CCC—defined by the presence of specific symptoms, such as fatigue, post-exertional malaise (PEM), sleep dysfunction, pain, two or more neurological/cognitive manifestations, and symptoms from at least two of the following categories: autonomic, neuroendocrine, and immune manifestation. The illness must persist for 6 or more months. Careful history-taking, physical examination, and clinical tests are needed to exclude other illnesses [7].

Studies have found elevated levels of autoantibodies (AABs) targeting adrenergic and muscarinic receptors in some ME/CFS patients, particularly M3, M4, β1, and β2 receptors [4,8]. Further research is needed to find proof that there is not only a significant clinical and pathophysiological overlap, but that shared therapeutic possibilities of ME/CFS and PCC could also exist. The clinical and biochemical similarities and differences in PCC and ME/CFS are displayed in Supplementary Table S1 [9,10]. Despite growing knowledge about the mechanisms of PCC, unfortunately, there is limited evidence-based data on effective curative therapies available today [11,12].

In this observational study, we aim to present how the wellbeing of our ME/CFS patients develops during and after extracorporeal PE + IVIG treatments. There are assumed to be three main underlying mechanisms of triggering ME/CFS that can be influenced by PE and IVIG-administration [6]:

1. Infection-induced: Infection functions as the initial trigger for a B-cell-mediated immune response.
2. Vascular and autoantibody-mediated dysfunction: GPCRs targeting antibodies may cause endothelial dysfunction, impaired neurovascular control, and autonomic small nerve fiber involvement. The clinical manifestations of this are impaired venous return, preload failure, and arteriovenous shunting, ultimately contributing to blood flow dysregulation and exertion-induced tissue hypoxia.
3. Secondary compensatory mechanisms: This involves compensatory adaptations, including increased sympathetic tone and metabolic shifts aimed at maintaining energy supply. These adaptations further contribute to the clinical presentation and symptomatology of ME/CFS.

There are numerous potential therapeutic strategies targeting these pathways [6]:

1. Interfere with the pathological immune response in the following ways [6]:
  - B-cell depletion therapy (anti-CD20 antibody) [13];
  - Cytotoxic drugs (cyclophosphamide);
  - Modulate plasma cell survival factors (Anti-BAFF antibody);
  - Plasma cell inhibition (Anti-CD38 antibody, proteasome inhibition);
  - Immunoglobulin manipulation (neonatal fragment crystallizable receptor (FcRn) targeting, immunoadsorption, IVIGs). Human immunoglobulins are meant to have an immunomodulatory and immunosuppressive effect, even at a low dosage [14].
2. Address vascular dysregulation, including endothelial dysfunction, arteriovenous shunting, impaired autoregulation. One study demonstrated improvement in endothelial dysfunction after plasmapheresis in critically ill patients with disseminated intravascular coagulation (DIC) [15].
3. Support the patient’s compensatory adaptation by pacing therapy [16] or by cognitive techniques [6].

In this study, all three major potential therapeutic strategies have been addressed: firstly, by applying plasmapheresis for improving endothelial dysfunction and platelet function; secondly, in terms of administering IVIG to regulate the derailed, inadequate immune response; and thirdly by performing pacing therapy to support the compensatory adaptation mechanisms.

Even if there is no reliable research data, up to the current date, which suggests that PE could improve clinical outcomes by extracting the microclots [17] that have been identified in PCC and ME/CFS patients, it has been proven that PE can reduce IgG levels [18] and can improve endothelial dysfunction and platelet function [15]. Given the better availability and lower cost of PE than immunoadsorption, positive results with a PE technique could provide a treatment option even for lower-income countries in the demanding need for ME/CFS therapy.

## 2. Materials and Methods

### 2.1. Study Protocol

Adult patients (≥18 years) with ME/CFS and elevated autoantibodies (against β1/β2-adrenergic receptors (ADRB1/2) and/or muscarinic receptors M3/M4 (CHRM3/4)), measured between January 2023 and September 2024, were enrolled in this pilot study at a Swiss tertiary care hospital. The patients were recruited at the Outpatient Clinic of the Department Internal Medicine via long COVID consultation. The 3 departments involved were as follows: the Outpatient Clinic of Internal Medicine (enrolling, initial diagnostics), the Division of Psychosomatics (psychometrics), and the Department of Nephrology and Transplant Medicine (PE, IVIG).

The patients went through a comprehensive work-up before being referred to plasmapheresis. They had been diagnosed with ME/CFS according to the Canadian Consensus Criteria. Other possible causes that could mimic ME/CFS-symptoms had been ruled out through cardiac testing by echocardiography, and in some cases also bicycle ergometry or cardiac MRI. They had undergone testing for early-onset neurodegenerative diseases by functional testing with the Montreal Cognitive Assessment (MoCA) and/or neuroimaging—cerebral computed tomography (CT) or cerebral magnetic resonance imaging (cMRI) scans—to rule out demyelinating diseases. Functional pulmonary testing by body plethysmography and carbon monoxide (CO) diffusing capacity testing had been performed, and in a few cases chest imaging had also been employed to assess possible pulmonary embolism, as well as sleep apnea screening in order to also rule out this possibility. Every patient completed a 6 min walk test (6MWT) and a Schellong test as baseline assessments. Some patients had been examined for possible gastrointestinal, rheumatological, and other neurological disorders.

Prior to the referral for PE, the patients were individually treated with various conservative therapeutic measures: professionally instructed pacing therapy, physiotherapy, psychotherapy, tricyclic antidepressants, serotonin–norepinephrine reuptake inhibitors (SNRIs), selective serotonin reuptake inhibitors (SSRIs), H1-antihistaminergic agents, ritalin, antiepileptics, naloxone, and in some cases even inpatient rehabilitation.

The inclusion criteria included being clinically diagnosed with ME/CFS with elevated GPCRs. Other pilot studies had shown the effects of repeated immunoadsorption on ME/CFS patients [2]; therefore, we decided to perform PE with IVIG infusions, as in the case of Kiprov [11], as an off-label therapy. This method could also remove the autoantibodies and may have been able to remove the microclots as well. New research implies that, in ME/CFS cases, a dysregulation of the coagulation system is present, and endothelial dysfunction and the downregulation of the complement machinery may be assessed with PE on a molecular level [19].

However, some concerns have been raised about the pathophysiological role of microclots in developing ME/CFS, implying that microclots do not play a significant role in pathogenesis [17].

For PE, we used the Spectra Terumo Optia Apheresis System (Terumo Deutschland GmbH, Zweigniederlassung Spreitenbach, Bodenäckerstrasse 3, CH-8957 Spreitenbach, Switzerland). For the exchange, we used albumin 5%, one times the plasma volume. A total of 4 treatments (on days 1 and 5, and at a 1 to 4 month interval) were performed. The autoantibodies were measured before and two weeks after the fourth (in two cases after the third) plasma exchange. At the Outpatient Clinic of Internal Medicine, the patients received standardized tests for the assessment of physical function and symptoms in a standardized manner (interdisciplinary workflow, Table 1).

Among the 7 enclosed patients, 5 had peripheral venous access inserted directly before the PE. Two of them needed a central venous catheter for the PE. All the therapies were performed in an outpatient setting.

At the end of every PE session, the patients additionally received 2 g of Octagam^®^ (Octapharma AG Seidenstrasse 2, CH-8853 Lachen, Switzerland) intravenously, independently of their body weight. All patients were regularly monitored at Long COVID consultations during and after the series of 4 plasma exchange therapies. All the collected data were documented in the patient management system (PMS) of the hospital.

The ISI (Insomnia Severity Index), FSS (Fatigue Severity Scale), HADS (Hospital Anxiety and Depression Scale), and EQ-5D-5L (European Quality of Life 5 Dimensions 5 Level Version) questionnaires were applied to assess the symptoms of the patients through standardized methods. The EQ-5D-5L questionnaire was developed by the EuroQol Group and it is a well-studied tool for assessing quality of life. The EQ-5D-5L descriptive system comprises five dimensions (mobility, self-care, usual activities, pain/discomfort, and anxiety/depression). Each dimension has five response levels: no problems, slight problems, moderate problems, severe problems, and unable to/extreme problems. The index value is calculated by deducting the appropriate weights from 1, the value for full health [20].

### 2.2. Study Procedure and Data Collection

All four autoantibodies were measured two times per patient over the course of the study at the following measurement time points: a baseline before starting PE and two weeks after the last PE. Clinical outcomes—consisting of the 6MWT, Schellong test, and psychometric assessments with the ISI (insomnia), FSS (fatigue), HADS (depression and anxiety), and EQ-5D-5L (quality of life) questionnaires—were measured three times, at T1, T2, and T3, over the course of the study. T1 was before the first PE, T2 was 2 weeks after the second PE, and T3 was 2 weeks after the last PE. See Table 2 for the psychometric properties of the validated questionnaires used in this study.

### 2.3. Statistical Analyses

Concerning a potential main trial, the study design required a total of 56 patients for an expected 80% statistical power based on an a priori power analysis calculated with the SIMR package v.1.0.7 using Monte Carlo simulations [27]. The power analysis was based on a multilevel model (MLM) with two measurement time points for each patient and each of the two investigated variables, namely antibody concentration and clinical outcome measures. We expected a medium to large effect size R^2^ of 0.16, as comparable studies investigating the effect of plasma exchange treatments in ME/CFS patients with elevated β2-adrenergic receptor autoantibodies [28] and adrenergic dysfunction in ME/CFS patients [29] reported medium to large effect sizes. Cocks and Torgerson (2013) recommended having at least 9% of the sample size of the main planned trial when conducting a pilot study, resulting in 5 patients for the present pilot study [30].

Statistical analyses were performed using the R Project for Statistical Computing v4.4.2 [31]. After inspecting the incomplete data using the VIM package v6.2.2 [32], we imputed missing values with the MICE package v3.17.0 based on the multivariate imputation using the chained equations algorithm for the missing at random (MAR) scenario [33]. After imputation, we evaluated the imputed data using convergence diagnostic tools such as trace and density plots. Estimates of model parameters and weights obtained in the imputed datasets were further pooled over all imputations according to Rubin’s rule [34]. All statistical models were two-sided and based on a significance level of 5% (alpha = 0.05) [35].

To investigate the association between repeat-measured antibodies (predictors) and clinical outcomes (endpoints), we created a MLM using the LME4 package v1.1-36 [36]. MLMs are superior to repeated measures correlation (rmcorr) analysis when dealing with small sample sizes and data models with both random intercepts and slopes [36]. We considered Bonferroni correction for multiple testing [37]. All MLM comparisons were visualized through heatmap plots and the use of the GPLOTS package v3.2.0 [38].

We further compared clinical outcomes across the three measurement time points with one-way (time) repeated measures analysis of variance (rmANOVA). Creating linear mixed-effects models (LMMs) using the LME4 package v1.1-36, we controlled random subject effects [36]. We used Mauchly’s test of sphericity to test clinical outcome data for normality [35], and visualized data through box plots generated with the GGPLOT2 package v3.5.1 [39]. Concerning the EQ-5D-5L questionnaire, we used the eq5d package v0.15.2 to calculate the index score based on a reference German population, as there was no Swiss population available [40,41].

## 3. Results

This pilot study, with a statistical power of 10%, included seven patients, with five being male and two being female. Their mean age was 45 ± 10.13 years, with a range between 30 and 57 years.

**Figure 1:**
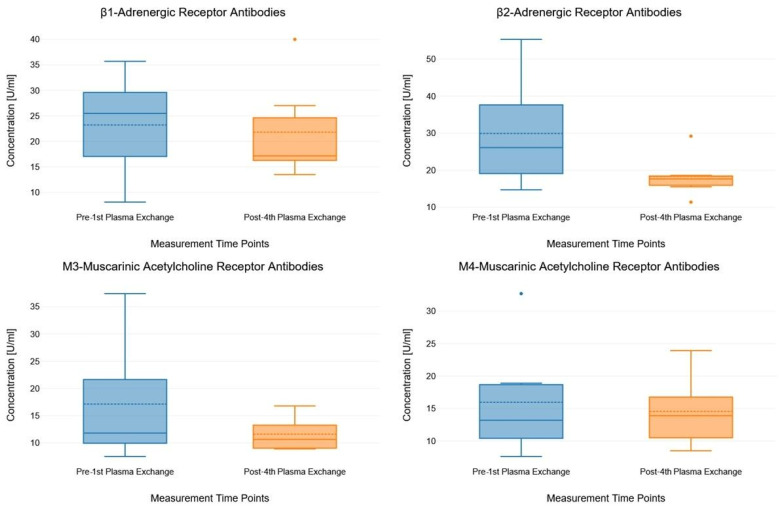
Box plots showing the concentration levels of the antibodies measured pre- and post-plasma exchange. The box plots consist of measurement time points (pre- and post-plasma exchange, x-axis) and antibody concentration (y-axis). In each box plot, the solid lines indicate median values and the dashed lines indicate mean values.

Figure 1 illustrates the concentration levels of all four antibodies measured before the first and after the fourth PE using box plots. None of the antibodies showed a significant change in concentration levels between the two measurement time points.

Due to time constraints, two patients prematurely terminated the study after T1 and T2, meaning we are lacking two-thirds and one-third of their clinical outcome data, respectively. One of these two patients lacked antibody data at the end of therapy. The missing values of these patients were further imputed and included in the analyses. Across all MLM comparisons between antibodies and clinical outcomes, there were two statistically significant associations: higher concentrations of β2-adrenergic receptor antibodies (R^2^ = 0.32, t(504) = −2.46, *p* = 0.014 *) and M3-muscarinic acetylcholine receptor antibodies (R^2^ = 0.46, t(110) =−3.17, *p* = 0.002 **) were both relevant predictors of a lower and therefore worse EQ-5D-5L index score. In other words, per 1 U/mL increase in the concentration levels of β2-adrenergic receptor antibodies or M3-muscarinic acetylcholine receptor antibodies, the EQ-5D-5L index score [−0.59 to 1] decreased by 0.01 (0.63%) or 0.02 (1.26%), respectively.

**Figure 2:**
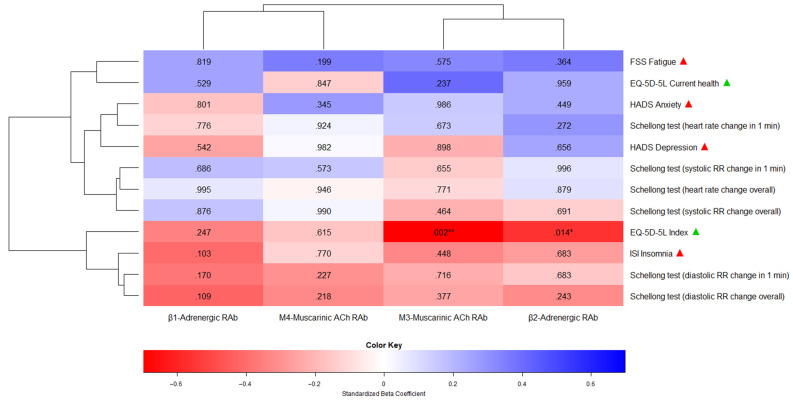
A heatmap plot showing the association between repeat-measured antibodies and clinical outcomes (T1 and T3). The heatmap consists of predictor variables (antibodies, x-axis), endpoints (clinical outcomes, y-axis), standardized beta coefficients (red for negative coefficients between 0 and −1, blue for positive coefficients between 0 and +1), and *p*-values (inside the heatmap plot). The beta coefficients indicate the direction and strength of the association between each predictor and endpoint, while the *p*-values indicate statistical significance. The terms “ACh” and “RAb” as predictor labels stand for “Acetylcholine” and “Receptor Antibody”, respectively. Next to the endpoints, the green and red triangles show whether higher scores indicate better or worse outcomes, respectively. The number of asterisks corresponds to the *p*-value range: * (*p* < 0.05), ** (*p* < 0.01), and *** (*p* < 0.001).

However, these results did not remain statistically significant after Bonferroni adjustment for multiple testing. Supplementary Table S2 shows the detailed results of all the MLM comparisons between antibodies and clinical outcomes measured at T1 and T3, while Figure 2 illustrates all the MLM comparisons using a heatmap plot.

**Figure 3:**
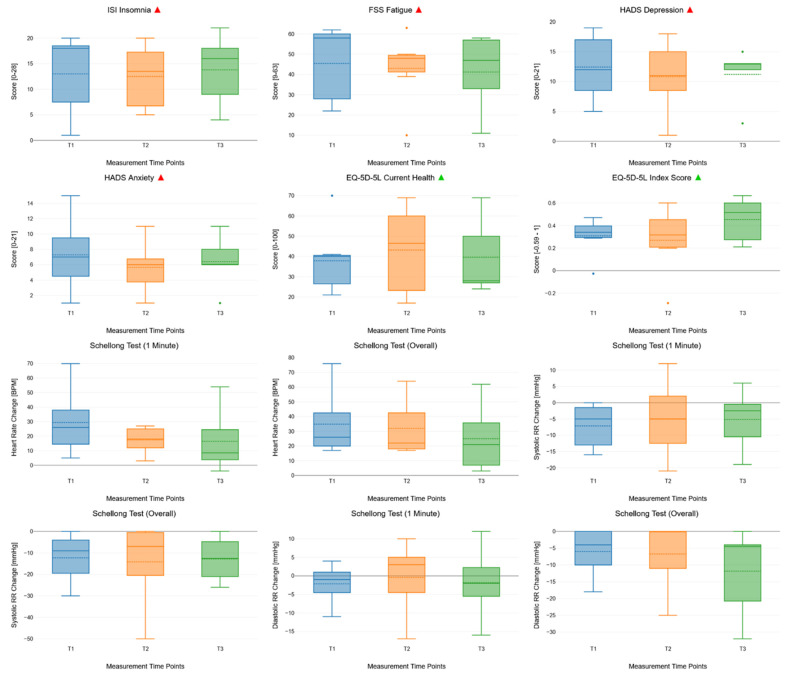
Box plots showing the changes in repeat-measured clinical outcomes across all measurement time points (T1, T2, and T3). The box plots consist of measurement time points (T1, T2, and T3, x-axis) and endpoints (clinical outcomes, y-axis). In each box plot, the solid lines indicate median values and the dashed lines indicate mean values. Next to the main title of the boxplots, the green and red triangles show whether higher scores indicate better or worse clinical outcomes, respectively.

There were no statistically significant improvements in clinical outcomes across T1, T2, and T3. However, we found trends towards the improvement of health-related quality of life as measured with the index score of the EQ-5D-5L questionnaire. See Supplementary Table S3 for the detailed results of the rmANOVA of clinical outcomes over the course of the study. Figure 3 shows the unimputed clinical outcomes over the course of the study, measured at T1, T2, and T3, using box plots.

## 4. Discussion

The aim of the current study was to determine whether the reduction in AAB levels in this specific patient population (patients with PCC, that fulfill the criteria for ME/CFS, with elevated AABs) has a potentially beneficial clinical effect. In this pilot study, we focused on the central entity, ME/CFS, regardless of the presumed triggering factor. Numerous standardized follow-up tests (the Schellong test, 6MWT with Borg Score, EQ-5D-5L Health VAS, EQ-5D-5L, HADS Anxiety, HADS Depression, ISI, FSS, and IES-R) were performed repeatedly to assess the potential effect on the studied population.

This study identified a significant association between elevated levels of β2-adrenergic receptor antibodies/M3-receptor antibodies and clinical outcomes in the studied subpopulation. Per 1 U/mL increase in the concentration levels of β2-adrenergic receptor antibodies or M3-muscarinic acetylcholine receptor antibodies, the EQ-5D-5L index score [−0.59 to 1] decreases by 0.01 (0.63%) or 0.02 (1.26%), respectively. Higher concentrations of these autoantibodies are associated with a deterioration in quality of life, highlighting their potential role in the pathophysiology of this condition. Most patients (five of a total of seven) reported a subjective benefit after the first PE + IVIG therapy; however, it could be observed that many of them were crushed after the second round. This deterioration of clinical status led to the premature termination of therapy in three cases. Over the course of the four treatments, most patients experienced a gradual improvement in their symptoms.

Another study group using immunoadsorption (IA) analyzed blood samples directly after the fourth procedure as well. Here, there was shown to be a more significant reduction in AAB titers (mean ADRB2 77%) at this time point; however, 4 weeks after the first IA (and approximately 18 days after the last IA), they detected milder changes in the AAB titers (6–19% reduction) [28]. However, there was no correlation between AAB levels and response. Thus, mechanisms beyond simple autoantibody depletion are likely responsible for the improvement observed in a subset of patients [1].

A comparable cohort study to our investigation, considering 27 patients with long COVID, was conducted with INUSpheresis, administering two treatments at an interval of 3 weeks [42]. Blood samples were collected before initiating and after the second apheresis. After the treatment, autoantibodies against β1- and β2-adrenergic receptors were decreased by 33% and 28%, respectively, whereas autoantibodies against M3- and M4-anticholinergic receptors were reduced by 48% and 39%, respectively [42].

The milder, and in our case non-significant, AAB titer reduction in contrast to the above-cited experiments could be a consequence of the different measuring time points (directly after the last extracorporeal therapy vs. 2 weeks after) or the differing modalities (PE vs. IA vs. INUSpheresis).

A retrospective case–control study compared 3 groups of 10 patients each: Group 1 received IVIG in addition to supportive treatment, Group 2 was treated with inhaled glucocorticoids (budesonide,) and Group 3 was on supportive measures. The patient group, that received IVIG in addition to supportive measures implicated the greatest benefit, measured with modified ISARIC scores [43]. This element (IVIG) could have contributed to the statistically detectable positive trend in our results.

The main strength of this study was the precise patient selection that ruled out all other possible conditions that can mimic ME/CFS. Before the recruitment for PE and IVIG combination therapy, all patients underwent the available conservative therapy options (physiotherapy, rehabilitation, pharmaceuticals), and elevated AABs were inclusion criteria. AAB measurements were conducted before the first and two weeks after the last PE + IVIG treatments. There is no other study in the literature that has investigated the therapeutic effect of the combination of PE and IVIG in ME/CFS cases. This choice of therapeutic measures was based on the positive results of theoretical (IVIG: [44,45,46]; PE: [47]) and clinical (IVIG: [43]; PE: [28,42]) publications in the field.

Lacking consensus statements or clinical guidelines, at this early stage of clinical research the study designs are hardly comparable. A review article from 2024 [48] including 18 representative studies demonstrates the wide range of researched therapeutic methods for PCC. Seven categories for potential PASC treatment have been identified: antihypertensive/ADHD treatment, antioxidant, antidiabetic, immunomodulator (including IVIG), statins, and the category “other”, including plasmapheresis. Metformin (phase 2–3 RCTs) [49] and low-dose naltrexone (large cohort studies) [50] stand out with the strongest evidence. Dexamethasone and remdesivir administration in the acute phase of COVID-19 resulted in a lower percentage of PCC patients in a single-center prospective observational study of 1966 patients [51].

Today, the role of GPCR-AABs in the pathogenesis of ME/CFS is not completely understood. A former study compared the AAB levels of a total of 80 patients composed of the following subgroups: post-COVID ME/CFS patients, PCS but non-ME/CFS patients, healthy SARS-CoV-2-naive (HC) and post-COVID healthy (PCHC) individuals [4]. The authors stated that certain individuals in the HC and PCHC groups exhibited higher levels of AABs compared to patients with PCS, despite being asymptomatic. This observation suggests that elevated AAB concentrations alone may not be inherently pathogenic. Rather, the pathogenic potential of AABs likely depends on additional factors, such as their functional activity, receptor interactions, and the broader physiological context in which they occur. In the same study, the researchers found a positive correlation of AAB levels with symptom severity within the PCS groups [4]. AAB levels against β2-adrenergic -AAB and M4-muscarinic acetylcholine receptor antibodies (CHRM4) decreased in clinical responders to rituximab, suggesting a link to disease activity [52]. Elevated vasoregulatory GPCR AABs correlate with fatigue and muscle pain in post-infectious ME/CFS patients. In contrast, patients with non-infection-triggered ME/CFS exhibited fewer and distinct correlations [53]. These findings support an association between AABs targeting ADRs and CHRMs and ME/CFS [4]; however, other researchers could not find a connection between AAB titers and disease activity [8]. Considering this correlation, by performing plasma exchange, the primary aim was to reduce autoantibody levels. There is a lack of hard evidence that PE can extract microclots, or that they contribute to the symptoms [17]; therefore, the study group did not investigate this topic in the current research.

### 4.1. Safety

Safety is a major concern for any medical intervention. A retrospective, single-center review study of centrifuge-based therapeutic plasma exchange [54] including 1219 PE treatments in 145 patients reported the following common complications: depletion coagulopathy (47.6%), hypocalcemia (44.1%), and hypokalemia (36.6%). The detected coagulopathy was a laboratory finding, no bleeding complications occurred. Our study group controlled the platelet count and INR before every session, in order to avoid the aggravation of any underlying coagulopathy. Furthermore, blood gas samples were analyzed after each hour of PE for electrolyte abnormalities, and if needed, substitution was administered.

Previous studies have reported overall complication rates in the range of 11–18%, and major complications (cardiovascular events, respiratory events, anaphylactoid reaction, hemorrhage, sepsis) in 0.025–4.75% of all sessions [55,56,57]. Hypotension, complications during the catheter placement procedure, and allergic reactions are other frequent complications. In our study, one patient out of seven (14%) developed a catheter-related infection, and therefore the catheter had to be prematurely extracted. This complication rate is higher compared to the result of the cited review [54] (4.1%), probably disproportionately, due to the lower statistical power of our low-number study.

### 4.2. Limitations

It should be stated that the studied patient population was highly prefiltered and specific, in terms of representing solely patients with ME/CFS as defined by the CCC, with elevated GPCRs, who had no satisfying response to previous conservative therapeutic measures for ME/CFS. Therefore, our results allegedly do not apply for every ME/CFS case, and should be seen as a hypothesis-building pilot study. In the past few years, in the field of ME/CFS and PCC research, many contradictory articles have been published. Some of them have argued for the important role of AABs in the pathogenesis of ME/CFS, seeing potential treatment options in the prohibition of their synthesis or in their removal [6,15,45,58,59,60]. Other groups of researchers have also observed the presence of activated B-cells or GPCRs in ME/CFS cases, whereby there was no correlation with the clinical presentation of the patients [61,62].

Besides the low number of patients, one limitation of this pilot study is that our sample consists of five male (71%) and two female (29%) patients, which is not consistent with the male–female ratio of 45:55 found in a broad meta-analysis of the prevalence of ME/CFS patients. The age distribution of our sample (45 ± 10.13), however, is comparable to the results (40.0 ± 9.9 years) of the meta-analysis [63].

Working with ME/CFS patients might be challenging. The core symptom, post-exertional malaise (the so-called “crushes”), could interfere with adequate data collection, even if the study group works within a well-established study protocol. Some of the patients suffered from recurring post-exertional malaise after the conduction of the 6MWT. Therefore, our study group decided to cease the 6MWT for the wellbeing of our patients. Consequently, the 6MWT dataset was not eligible for statistical analyses. ME/CFS itself, due to its main clinical manifestation being characterized by exertion intolerance and post-exertional malaise, poses a huge challenge for adequate data collection. During their crushes, ME/CFS patients sometimes have to cancel consultations or blood tests because of the major malaise. This might have negatively impacted the data collection. One patient had to be excluded, because he/she lacked baseline and follow-up clinical outcome data. Two patients were analyzed after data imputation.

## 5. Conclusions

This study identified a significant association between elevated levels of β2-adrenergic receptor antibodies/M3-receptor antibodies, and clinical outcomes in patients with ME/CFS. The data indicate that higher concentrations of these autoantibodies are associated with a deterioration in quality of life, highlighting their potential role in the pathophysiology of this condition.

The combination of PE and IVIGs not only aims to alleviate symptoms associated with autoantibody-mediated dysfunction, but also to provide a protective effect against potential infections.

Our findings suggest that additional therapeutic strategies may be warranted for these patients. Specifically, B-cell depletion therapy could further reduce the production of these harmful autoantibodies and enhance overall treatment efficacy. Integrating plasma exchange, human immunoglobulin administration, and eventually B-cell-targeted therapies could possibly significantly improve the quality of life of individuals suffering from ME/CFS. Future large-scale randomized controlled trials are needed to validate these therapeutic strategies and optimize management approaches for this complex and challenging condition.

## Figures and Tables

**Table 1:** Interdisciplinary workflow, patient management, and consultations during study.

1.	Outpatient Clinic of Department of Internal Medicine Consultation of Long COVID	Relevant laboratory results: β1-adrenergic receptor antibody, β2-adrenergic receptor antibody, M3-muscarinic acetylcholine-receptor-antibody, M4-muscarinic acetylcholine-receptor-antibody. Standardized tests: Schellong test, 6MWT with Borg Score, echocardiography, pulmonal functional tests, psychiatric evaluation, EQ-5D-5L Health VAS, EQ-5D-5L, HADS Anxiety, HADS Depression, ISI, FSS, IES-R. If the detected antibodies showed a relevant elevation, the non-responder patients to conservative therapy were assigned to the apheresis consultation.
2.	Apheresis Consultation at Clinic of Nephrology and Transplant Medicine	The patients were informed about all side effects and complications of the plasma exchange and albumin application.
3.	1st Plasma Exchange	A clinical visit to Nephrology.
4.	2nd Plasma Exchange after 5 Days	A clinical visit to Nephrology.
5.	Back to Long COVID Consultation within 2 Weeks	A clinical visit to and standardized tests at Internal Medicine.
6.	3rd Plasma Exchange after 1 Month	A clinical visit to Nephrology.
7.	Back to Long COVID Consultation within 8 Weeks	A clinical visit to and standardized tests at Internal Medicine.
8.	4th Plasma Exchange after 1–4 Months	A clinical visit to Nephrology.
9.	Back to Long COVID Consultation within 2 weeks	A clinical visit, laboratory and standardized tests, and follow-up.

**Table 2:** Psychometric properties of validated questionnaires completed at three measurement time points (T1, T2, T3).

Outcome	Questionnaire/Reference	Score Building/Range/Cut-Off/(Sub)Scale
Insomnia	ISI (Insomnia Severity Index)/[21,22]	Sum score 0 to 28/≥15/global
Fatigue	FSS (Fatigue Severity Scale)/[23,24]	Sum score/9 to 63/≥36/global
Depression and anxiety	HADS (Hospital Anxiety and Depression Scale)/[25,26]	Sum score/0 to 21/≥8/depression Sum score/0 to 21/≥8/anxiety
Health-related quality of life	EQ-5D-5L (European Quality of Life 5 Dimensions 5 Level Version)/[20]	Visual analogue scale score/0 to 100/no cut-off/ current health Index score −0.59 to 1/no cut-off/quality of life consisting of five dimensions: mobility, self-care, usual activities, pain/discomfort, anxiety/depression

## Data Availability

Research data are available at: https://dx.doi.org/10.6084/m9.figshare.29142482.

## Supplementary Materials

The following supporting information can be downloaded at: https://www.mdpi.com/article/10.3390/jcm14113802/s1, and https://dx.doi.org/10.6084/m9.figshare.29142482.

## Abbreviations

The following abbreviations are used in this manuscript:

6MWT	6 min walk test
ACTH	adrenocorticotropic hormone
ADRs	adrenergic receptor antibodies
ADRB2	adrenergic 2-receptor antibody
CHRM3	M3-muscarinic receptor antibody
CHRM4	M4-muscarinic receptor antibody
AT1-R	angiotensin-1 receptor
CO	carbon monoxide
CRH	corticotropin-releasing hormone
CT	computed tomography
DIC	disseminated intravascular coagulation
EBV	Epstein–Barr virus
EQ-5D-5L	European Quality of Life 5 Dimensions 5 Level Version
ETA-R-AAB	Endothelin-1 type A receptor antibody
FcRn	neonatal fragment crystallizable receptor
FSS	Fatigue Severity Scale
GPCRs	G-protein-coupled receptors
HADS	Hospital Anxiety and Depression Scale
IA	immunoadsorption
IgG	immunoglobulin G
IGF-1	Insulin-like growth factor 1
IGF-2	Insulin-like growth factor 2
ISI	Insomnia Severity Index
IVIG	intravenous immunoglobulin
LMM	linear mixed-effects model
ME/CFS	myalgic encephalomyelitis/chronic fatigue syndrome
MLM	multilevel model
MoCA	Montreal Cognitive Assessment
PASC	postacute sequelae of SARS-CoV-2 infection
PCC	long COVID/post-COVID-19 condition
PE	plasmapheresis/plasma exchange
PEM	post-exertional malaise
POTS	postural orthostatic tachycardia syndrome
SARS-CoV-2	severe acute respiratory syndrome coronavirus 2
SNRIs	serotonin–norepinephrine reuptake inhibitors
SSRIs	selective serotonin reuptake inhibitors
alpha1/2-AdR-AAB	adrenergic α1/2-receptor antibody
