# Supplementary material for: The Effect on Quality of Life of Therapeutic Plasmapheresis and Intravenous Immunoglobulins on a Population of Myalgic Encephalomyelitis/Chronic Fatigue Syndrome Patients with Elevated β-Adrenergic and M3-Muscarinic Receptor Antibodies—A Pilot Study
Source: J Clin Med. 2025 May 29;14(11):3802. doi: 10.3390/jcm14113802 (PMC12155665; doi:10.3390/jcm14113802)
Supplement: Supplementary file 1 [file jcm-14-03802-s001.zip › jcm-3587984-supplementary.pdf]

Journal of
Clinical Medicine

SUPPLEMENTARY MATERIAL

 https://doi.org/10.3390/xxxxx

Supplementary Table S1. Comparison of similarities of ME/CFS and PCC symptoms and characteristics findings. The table is based on the review article of A. L. Komaroff and W. I. Lipkin [1]

Main symptom by category	ME/CFS	PCC
Cognitive impairment	attention, reaction time, which worsens after physical/cognitive exertion	memory, attention, executive functions
	cerebral hypometabolism	up to 10 months*
	reduced cerebral blood flow	reduced cerebral blood flow (up to 10 months post-infection)
Hypothalamic-pituitary growth hormon abnormalities	reduced peripheral CRH, ACTH, cortisol and IGF1 and IGF2 levels*	reduced peripheral CRH, ACTH, cortisol and IGF1 and IGF2 levels*
Autonomic dysfunction	a variety of autonomic testing showed impairments, autonomic dysfunction correlates with symptom severity*	similar findings to ME/CFS-group
Pain	lowered treshold	central sensitisation possible
	small fiber neuropathy	
Sleep disturbances	insomnia, non-restorative sleep*, irregular sleep patterns	chronic insomnia, hypersomnia, irregular sleep patterns
Autoantibodies	adrenergic α1 receptor antibody (gastrointestinal symptoms) [2] adrenergic α2- receptor antibody (gastrointestinal symptoms)[2]	adrenergic α1 receptor antibody [3] adrenergic β2 receptor antibody [3]
	Mas-receptor antibody against angiotensin-II T1 receptor Endothelin-1 type A receptor antibody (cognitive impairment) [2]	Mas-R antibody against angiotensin-II T1 receptor [3]
Clotting abnormalities	microclots, hyperactivated platelets	fibrin amyloid microclots, with entrapped pro-inflammatory molecules ↓ plasma Kallikrein, ↑ platelet factor 4, ↑ von Willebrand factor, marginally increased level of α-2 antiplasmin [4]

CRH= corticotropin releasing hormon, ACTH= adrenocorticotropic hormone, IGF-1= Insulin-like growth factor 1, IGF-2= Insulin-like growth factor 2
* existing confirming as well as negative studies

[1].

[1] A. L. Komaroff und W. I. Lipkin, „ME/CFS and Long COVID share similar symptoms and biological abnormalities: road map to the literature", *Front. Med.*, Bd. 10, S. 1187163, Juni 2023, doi: 10.3389/fmed.2023.1187163.
[2] H. Freitag *u. a.*, „Autoantibodies to Vasoregulative G-Protein-Coupled Receptors Correlate with Symptom Severity, Autonomic Dysfunction and Disability in Myalgic Encephalomyelitis/Chronic Fatigue Syndrome", *J. Clin. Med.*, Bd. 10, Nr. 16, S. 3675, Aug. 2021, doi: 10.3390/jcm10163675.
[3] C. Szewczykowski *u. a.*, „Long COVID: Association of Functional Autoantibodies against G-Protein-Coupled Receptors with an Impaired Retinal Microcirculation", *Int. J. Mol. Sci.*, Bd. 23, Nr. 13, S. 7209, Juni 2022, doi: 10.3390/ijms23137209.
[4] A. Kruger *u. a.*, „Proteomics of fibrin amyloid microclots in long COVID/post-acute sequelae of COVID-19 (PASC) shows many entrapped pro-inflammatory molecules that may also contribute to a failed fibrinolytic system", *Cardiovasc. Diabetol.*, Bd. 21, Nr. 1, S. 190, Sep. 2022, doi: 10.1186/s12933-022-01623-4.

Supplementary Table S2. Pooled results of the associations between antibodies and imputed clinical outcomes using a multilevel model (MLM) with point estimate values representing non-standardized MLM coefficients. Up to two decimal places were added to the figures when rounding issues occurred.

Independ. variable / predictor	Depend. variable / endpoint	Point estimate	Lower 95% CI	Upper 95% CI	Std. error	T-stat.	Degrees of freedom	P-val.	R2
**β1-Adrenergic Receptor Antibody**	ISI Insomnia	-0.30	-0.67	0.06	0.19	-1.63	2910.66	.103	0.17
	FSS Fatigue	0.16	-1.31	1.63	0.70	0.23	17.10	.819	0.04
	HADS Anxiety	-0.03	-0.30	0.23	0.14	-0.25	86.63	.801	0.02
	HADS Depression	-0.11	-0.47	0.25	0.18	-0.61	64.73	.542	0.06
	EQ-5D-5L Index	-0.01	-0.02	0.01	0.01	-1.16	156.69	.247	0.12
	EQ-5D-5L Current health	0.39	-0.84	1.62	0.61	0.63	52.83	.529	0.06
	Schellong test								
	Heart rate change (1 min)	-0.20	-1.57	1.17	0.70	-0.28	424.78	.776	0.01
	Heart rate change (overall)	0.004	-1.32	1.33	0.67	0.01	1300.64	.995	0.003
	Systolic RR change (1 min)	0.11	-0.41	0.62	0.26	0.41	106.71	.686	0.03
	Systolic RR change (overall)	0.06	-0.71	0.83	0.39	0.16	68.47	.876	0.02
	Diastolic RR change (1 min)	-0.07	-0.45	0.31	0.16	-0.42	7.30	.683	0.06
	Diastolic RR change (overall)	0.44	-0.66	1.54	0.44	0.99	5.75	.364	0.09
**β2-Adrenergic Receptor Antibody**	ISI Insomnia	-0.07	-0.45	0.31	0.16	-0.42	7.30	.683	0.06
	FSS Fatigue	0.44	-0.66	1.54	0.44	0.99	5.75	.364	0.09
	HADS Anxiety	0.06	-0.10	0.23	0.08	0.76	72.77	.449	0.05
	HADS Depression	0.06	-0.21	0.33	0.12	0.46	12.24	.656	0.04
	EQ-5D-5L Index	-0.01	-0.02	-0.002	0.003	-2.46	504.26	**.014***	0.32

	EQ-5D-5L Current health	0.02	-0.99	1.04	0.48	0.05	15.92	.959	0.04
	Schellong test								
	Heart rate change (1 min)	0.49	-0.39	1.38	0.45	1.10	147.33	.272	0.08
	Heart rate change (overall)	0.07	-0.87	1.01	0.48	0.15	685.47	.879	0.01
	Systolic RR change (1 min)	0.00	-0.34	0.34	0.17	0.01	763.25	.996	0.004
	Systolic RR change (overall)	-0.09	-0.54	0.36	0.23	-0.40	342.55	.691	0.02
	Diastolic RR change (1 min)	-0.05	-0.28	0.19	0.11	-0.41	26.13	.683	0.02
	Diastolic RR change (overall)	-0.28	-0.75	0.19	0.24	-1.17	312.92	.243	0.11
**M3-Muscarinic Acetyl-choline Receptor Antibody**	ISI Insomnia	-0.18	-0.72	0.35	0.23	-0.80	7.23	.448	0.09
	FSS Fatigue	0.43	-1.34	2.21	0.74	0.59	6.40	.575	0.06
	HADS Anxiety	0.00	-0.27	0.27	0.13	0.02	48.48	.986	0.01
	HADS Depression	-0.02	-0.40	0.35	0.18	-0.13	17.53	.898	0.03
	EQ-5D-5L Index	-0.02	-0.02	-0.01	0.01	-3.17	110.29	**.002****	0.46
	EQ-5D-5L Current health	0.72	-0.50	1.94	0.60	1.21	30.06	.237	0.15
	Schellong test								
	Heart rate change (1 min)	0.29	-1.07	1.66	0.70	0.42	322.16	.673	0.02
	Heart rate change (overall)	-0.20	-1.57	1.17	0.70	-0.29	822.55	.771	0.01
	Systolic RR change (1 min)	-0.11	-0.60	0.38	0.25	-0.45	635.23	.655	0.02
	Systolic RR change (overall)	-0.25	-0.91	0.41	0.33	-0.73	249.11	.464	0.04
	Diastolic RR change (1 min)	-0.08	-0.55	0.39	0.21	-0.37	10.23	.716	0.04
	Diastolic RR change (overall)	-0.31	-0.99	0.38	0.35	-0.88	503.73	.377	0.06
**M4-Muscarinic Acetyl-choline Receptor Antibody**	ISI Insomnia	-0.08	-0.61	0.45	0.27	-0.29	216.60	.770	0.01
	FSS Fatigue	0.90	-0.48	2.28	0.69	1.30	68.50	.199	0.10
	HADS Anxiety	0.15	-0.17	0.47	0.16	0.95	411.64	.345	0.08
	HADS Depression	0.01	-0.40	0.41	0.21	0.02	155263.9	.982	0.0003
	EQ-5D-5L Index	-0.004	-0.02	0.01	0.01	-0.50	585.01	.615	0.03
	EQ-5D-5L Current health	-0.15	-1.63	1.34	0.75	-0.19	180.80	.847	0.01
	Schellong test								
	Heart rate change (1 min)	0.09	-1.70	1.87	0.91	0.10	96242.33	.924	0.001
	Heart rate change (overall)	-0.06	-1.80	1.68	0.89	-0.07	23644.78	.946	0.001
	Systolic RR change (1 min)	0.17	-0.43	0.78	0.31	0.56	905826.11	.573	0.03

Systolic RR change (overall)	0.01	-0.88	0.90	0.45	0.01	40299.40	.990	0.001
Diastolic RR change (1 min)	-0.30	-0.78	0.19	0.25	-1.21	609.36	.227	0.10
Diastolic RR change (overall)	-0.51	-1.33	0.30	0.42	-1.23	9418.34	.218	0.11

Supplementary Table S3. Pooled results of one-way (time) repeated-measures analysis of variance (rmANOVA) of imputed clinical outcomes across three measurement time points (T1, T2, T3).

Outcome	Effect	Degrees of freedom (nom.)	Degrees of freedom (denom.)	Mauchly's test of sphericity	F	p	R2
**ISI Insomnia**	Time	2	13.44	Equal variances	0.20	0.818	0.03
**FSS Fatigue**	Time	2	24.43	Equal variances	0.41	0.667	0.04
**HADS Depression**	Time	2	49.56	Equal variances	0.48	0.621	0.03
**HADS Anxiety**	Time	2	293.17	Equal variances	0.65	0.521	0.04
**EQ-5D-5L Current health**	Time	2	26.89	Equal variances	0.38	0.686	0.05
**EQ-5D-5L Index score**	Time	2	1072.85	Equal variances	2.30	0.101	0.17
**Schellong test**							
**Heart rate change (1 min)**	Time	2	216.16	Equal variances	1.84	0.162	0.12
**Heart rate change (overall)**	Time	2	60.05	Equal variances	0.58	0.565	0.04
**Systolic RR change (1 min)**	Time	2	198.57	Equal variances	0.16	0.853	0.02
**Systolic RR change (overall)**	Time	2	612.6	Equal variances	0.06	0.945	0.01
**Diastolic RR change (1 min)**	Time	2	64.26	Equal variances	0.36	0.698	0.01
**Diastolic RR change (overall)**	Time	2	1232.92	Equal variances	1.59	0.204	0.10

Abbreviations: RR = Riva-Rocci / Blood pressure
